# Relationships between depression, health‑related behaviors, and internet addiction in female junior college students

**DOI:** 10.1371/journal.pone.0220784

**Published:** 2019-08-09

**Authors:** Shang-Yu Yang, Shih-Hau Fu, Kai-Li Chen, Pei-Lun Hsieh, Pin-Hsuan Lin

**Affiliations:** 1 Department of Occupational Therapy, College of Medical and Health Science, Asia University, Taichung, Taiwan; 2 Department of Healthcare Administration, College of Medical and Health Science, Asia University, Taichung, Taiwan; 3 Department of Acupressure Technology, Chung Hwa University of Medical Technology, Tainan, Taiwan; 4 Department of Nursing, College of Pharmacy and Health Care, Tajen University, Pingtung, Taiwan; 5 Department of Nursing, College of Health, National Taichung University of Science and Technology, Taichung, Taiwan; 6 Department of Health and Beauty, Shu Zen Junior College of Medicine and Management, Kaohsiung, Taiwan; International Telematic University Uninettuno, ITALY

## Abstract

**Introduction:**

Depressive emotions can lead to subsequent unhealthy behaviors such as Internet addiction, especially in female adolescents; therefore, studies that examine the relationships among depression, health‑related behaviors, and Internet addiction in female adolescents are warranted.

**Purpose:**

To examine (1) the relationship between depression and health-related behaviors and (2) the relationship between depression and Internet addiction.

**Method:**

A cross-sectional study design was adopted using a structured questionnaire to measure depression, health-related behaviors, and Internet addiction in female adolescents. The data were collected from students of a junior college in southern Taiwan using convenience sampling to select the participants. The questionnaire was divided into four sections: demographics, the Center for Epidemiologic Studies Depression Scale (CES-D), the Health Promoting Lifestyle Profile (HPLP), and the Internet Addiction Test (IAT).

**Results:**

The final sample comprised 503 female junior college students, with the participants mainly aged between 15 to 22 years (mean age = 17.30 years, SD = 1.34). Regarding the HPLP scores, the overall score, nutrition subscale score, and self-actualization subscale score were significantly and negatively associated with the CES-D depression score (*p* < 0.05–0.01). In other words, depression level was lower in students who exhibited more healthy behaviors, put more emphasis on dietary health, and had higher levels of self-admiration and confidence toward life. Regarding the IAT scores, the overall score and six domain scores were all positively associated (*p* < 0.01) to the CES-D depression score. In other words, the higher an individual’s Internet addiction score was, the higher her depression level was.

**Conclusions:**

The results confirmed the relationship between depression, health-related behaviors, and Internet addiction. The cultivation of health-related behaviors may help in lowering depressive symptoms. Teenagers with depression have higher risks of developing Internet addiction, and such addiction is likely to affect their daily functioning.

## Introduction

In Taiwan, 37.2% of the national adolescent population have experienced mood swings or episodes of depressive mood [1], and the number of adolescents affected by depression is increasing yearly [2]. During adolescence, in addition to the physical changes caused by puberty (e.g., hormonal changes), teens are faced with large-scale social and psychological developmental challenges (e.g., social relationships and school work). Accordingly, adolescence is a critical period for the development of depression and other psychiatric conditions [3, 4]. In addition to affecting physiology, psychology, social and familial relationships, and academic performance, depressive emotions can affect the health status of adolescents when they progress to adulthood [5, 6]. Previous studies have indicated that adolescent depression can result in the deterioration of sleep quality, daytime drowsiness, and increased obesity [7], and depression can develop into suicidal or self-harming ideation or behavior [8]. Teenagers with depressive tendencies also tend to have lower self-esteem, lower sense of well-being, and poorer social ability [9]; they are also more susceptible to developing addictions, including Internet addiction and substance abuse [10]. The negative effects of depressive emotions on adolescents, their families, and society are something that should not be overlooked. However, most studies on adolescent depression have only focused on understanding the roles of negative factors such as personal characteristics [11, 12] and family structures [13] in the development of teenage depression.

Some studies have indicated an association between health-related behaviors and depressive emotions in adolescents. For example, Kim, Yu [14] reported that drinking and smoking behaviors in adolescents are positively associated with depressive emotions. According to a past study [15], teenagers who have unhealthy dietary habits (e.g., frequently eating take-away food) are also more prone to developing depressive emotions. Teenagers with depressive emotions may have lower self-esteem [16], poorer interpersonal relationships, and poorer social ability [17]. Additionally, adolescents with depressive emotions are less willing to engage in physical activities such as participating in sports clubs or going to the gym [18]. However, research has also shown that the likelihood of adolescent depression occurrence can be lowered through education and cultivating health behaviors [19]. Health-promotion courses that were found to be effective in reducing the occurrence of adolescent depression include stress management courses, self-understanding courses, self-control courses, and interpersonal relationships courses [20, 21]. Therefore, to gain a more extensive understanding on the effects of health-related behaviors on depressive emotions, more research must be conducted that systematically examines the relationships between different health behavior dimensions and depressive emotions.

Due to the rapid development and popularization of the Internet in Asia, the prevalence of Internet addiction in the region has grown rapidly [22]. The results of research have also confirmed the relationship between Internet addiction and depressive emotions in Asian adolescents [16, 23–25]. For example, the studies conducted by Wu, Li [24] on 9,518 Hong Kong adolescents and Ha, Kim [23] on 452 Korean adolescents reported that depressive symptoms were positively associated with Internet addiction in adolescents. Another study conducted by Li, Hou [26] on 1,545 Chinese adolescents also reported that depression positively predicted adolescent Internet addiction. However, Park [27] asserted that other crucial factors may influence the development of Internet addiction in adolescents, such as performance of health-related behaviors; thus, the effects of health-related behaviors as potential confounding factors in adolescent depression must be examined. In addition, there are still different viewpoints on the “gender” issue of Internet addiction. A previous study [28] showed no differences between males and females in Internet disorder/addiction, while a recent study [29] reported that males were more prone to the development of Internet addiction than females. On the other hand, females are more likely to have negative emotions than males [30]. Therefore, the current study aimed at investigating whether negative emotions can also affect females’ use of the Internet.

Gender differences exist in the development of depression, and these differences are especially prominent during adolescence. The development of depressive emotions occurs earlier in female adolescents than in male adolescents, and the magnitude of depressive emotions tend to be more severe in female adolescents than in male adolescents [30]. Compared with depressive emotions in male teenagers, depressive emotions in female teenagers are more likely to lead to health problems such as obesity [31] and eating disorders [32]; moreover, depressive emotions in female teenagers are more likely to result in the development of unhealthy behaviors, such as Internet addiction [33, 34]. The high degree of depressive emotions and loneliness has been shown to have a strong relationship with Internet addiction, contributing to negative or dysfunctional social behaviors [35]. To explain this phenomenon, scholars have provided a theoretical model termed “compensated internet use” in which individuals use the Internet to escape reality and mitigate negative emotions [36]. Additional dispositional factors, such as the tendency for individuals higher in negative emotions to appear distant and aloof during social interactions in real living, may lead them to pursue satisfying interpersonal interactions online [36]. Therefore, the present study sought to examine the relationships between depressive emotions, health-related behaviors, and Internet addiction to gain a deeper understanding of the effects of depression on female adolescents. Accordingly, this study examined (1) the relationship between depression and health-related behaviors and (2) the relationship between depression and Internet addiction. The results of this study can serve as a reference for education authorities and parents to help alleviate the negative effects of depressive emotions in female teenagers.

## Methods

### Study design and subject recruitment

A cross-sectional study design was adopted using a structured questionnaire to measure depression, health-related behaviors, and Internet addiction in female adolescents. The data were collected from a junior college in southern Taiwan using convenience sampling to select the participants. The data collection period was from September to November 2018. The inclusion criteria were as follows: those who (1) were female, (2) could communicate orally in Mandarin or Taiwanese, and (3) could complete the questionnaire in written Chinese. The exclusion criteria were as follows: those who (1) did not complete the questionnaire, (2) were pregnant or already had children, or (3) were diagnosed with psychiatric disorders by a physician. Regarding procedures, the research assistants first explained the purpose of the study to the students. If a student agreed to participate, she was asked to sign the participant consent form before the questionnaire was distributed by the research assistants. Ethical approval for the study was obtained from the National Cheng Kung University Human Research Ethics Committee (No. NCKU HREC-E-106-108-2). Parental or guardian consent was obtained for students under 18 years.

### Research instruments

The questionnaire used in this study was divided into four sections: demographics, the Center for Epidemiologic Studies Depression Scale (CES-D), the Health Promoting Lifestyle Profile (HPLP), and the Internet Addiction Test (IAT). The demographics section collected participants’ age, body mass index (BMI), religion (i.e., whether the participant was religious), smoking status (i.e., whether the participant had smoked one or more cigarettes every day in the past 6 months), and alcohol drinking status (i.e., whether the participant had drunk alcohol every week in the past 6 months).

The CES-D that constituted the second section of the questionnaire was devised by Radloff in 1977. The tool was developed to measure depressive symptoms in the general population and to screen for populations at high risk for depression [37]. Currently, the CES-D is available in various languages [38]. The traditional Chinese version of the CES-D used in the current study was translated by Chien and Cheng [39]. The scale contains a total of 20 items, and the items measure the frequency of depressive symptoms in the past week. The scale adopts a 4-point Likert-type scoring system, with options ranging from 0 (less than 1 day) to 3 (5–7 days). The overall score ranges from 0 to 60, with a higher score indicating a higher level of depression. Typically, 16 points is taken as the cutoff point for depression; a score of 0–15 points indicates that the individual is not depressed, 16–20 points indicates that the individual is mildly depressed, 21–30 points indicates that the individual is moderately depressed, and a score higher than 30 points indicates that the individual is severely depressed [40]. One study [41] found that the Chinese version of the CES-D possesses good reliability and validity. In the present study, the Cronbach’s alpha coefficient for the overall CES-D score was .86.

The Chinese version of the HPLP was the third section of the questionnaire in this study. The HPLP was developed by Walker, Sechrist, and Pender in 1987 to evaluate the health-promotion lifestyle that an individual has exhibited in the past year. The scale contains 48 items spread over six behavioral domains. However, in the process of Chinese translation, the number of HPLP items was reduced to 40. One study found that the Chinese version of the HPLP possesses good reliability and validity [42]. The six behavioral domains of the Chinese version of the HPLP are as follows: (1) nutritional behavior (5 questions), evaluates whether the individual has healthy dietary habits and ideas; (2) health responsibility behavior (8 questions), examines whether the individual pays attention to his or her health, participates in health educational activities, and seeks professional help when necessary; (3) self-actualization (8 questions), evaluates whether the individual exhibits goal awareness, seeks personal development, and possesses a sense of self-awareness and self-fulfillment; (4) interpersonal support behavior (6 questions), examines whether the individual can achieve a sense of intimacy and closeness through meaningful interactions with family and close friends; (5) exercise behavior (4 questions), determines whether the individual performs physical activities that promote health; and (6) stress management behavior (9 questions), examines the individual’s understanding about the sources of stress and his or her ability to take stress-relieving actions. The scale adopts a 4-point Likert-type scoring system, with options ranging from 0 (*never*) to 3 (*always*); the total score ranges from 0 to 120, with a higher score indicating an individual exhibits more health-promotion behaviors. In the present study, the Cronbach’s alpha coefficient for the overall HPLP score was .95, and the scores for the six domains of nutrition, health responsibility, self-actualization, interpersonal support, exercise, stress management were .79, .88, .93, .94, .84 and .84, respectively.

The IAT used in the fourth section of the study questionnaire is a self-report inventory that was developed by Young (1998) to assess Internet addiction level. The IAT is available in various languages [43]. The scale contains 20 items, and six types of symptom complaint pattern can be identified based on the self-reported results: (1) Salience (5 items)—a higher score on salience-related items indicates that an individual is more likely to be preoccupied with the Internet and to exhibit less social interaction with others to the extent that he or she may lose interest in other activities and relationships in exchange for more online time. (2) Excessive use (5 items)—a higher score on excessive use-related items indicates that an individual is engaged in excessive Internet-use behaviors, and he or she is more likely to become depressed, panicked, or angry if Internet use is stopped. (3) Neglect work (3 items)—a higher score on neglect work-related items indicates that an individual views the Internet as an essential technology similar to a television or telephone, and his or her school or job performance and productivity will most likely be compromised due to the amount of time they spend online and their efforts in hiding their Internet usage from others. (4) Anticipation—a higher score on anticipation-related items indicates that an individual is likely to think about the things they would do when they are online again or anticipate being online again as soon as possible when they are not using the Internet. (5) Lack of control—a higher score on the lack of control-related items indicates that an individual is unable to manage his or her online time, and others may be unhappy or even complain about the amount of time the respondent spends online. (6) Neglect social life—a higher score on the neglect social life-related items indicates that the individual is likely to ignore social interactions in real life and in turn frequently use online relationships to cope with situational problems and reduce mental tension and stress. The scale adopts a 5-point Likert scoring system, with options ranging from 0 (*not applicable*) to 5 (*always*); the score ranges from 0 to 100, with a higher score indicating a higher level of Internet addiction. An overall score of 0–30 points reflects a normal level of Internet usage; a score of 31–49 points indicates a mild level of Internet addiction; a score of 50–79 points indicates a moderate level of Internet addiction; and a score of 80–100 points indicates a severe dependence upon the Internet. Psychometric studies have indicated that the IAT possesses good reliability and validity [43, 44]. In the present study, the Cronbach’s alpha coefficient for the overall IAT score was .93, and the scores for the six types of symptom of salience, excessive use, neglect work, anticipation, lack of control, neglect social life’s were .73, .79, .74, .78, .75, and .73, respectively.

### Statistical analysis

SPSS for Mac version 22 (IBM Corp., Armonk, NY) was used for data analysis in this study. First, descriptive statistical analyses were conducted to present the demographic characteristics of the research participants and obtain the means and standard deviations (SDs) for the CES-D, HPLP, and IAT. Second, the Pearson’s correlation coefficient (r) was used to investigate the correlation between IAT overall and each subscale score, HPLP overall score and each subscale scores, and the CES-D depression score. Third, a regression analysis was performed to examine the relationship between the CES-D and HPLP results. While controlling for the influence of demographic characteristics (i.e., age, BMI, smoking status, alcohol drinking status, and religion), the CES-D depression score was set as the dependent variable, whereas the HPLP overall score and six HPLP subscale scores were set as the independent variables. Subsequently, analysis of variance (ANOVA) and chi-squared tests (Tukey post hoc tests) were performed to examine if the three Internet addiction severity groups exhibited significant differences in terms of demographic characteristics and CES-D depression score. Fourth, a regression analysis was conducted to examine the relationship between the CES-D and IAT scores. While controlling for the influence of demographic characteristics and the HPLP overall score, the CES-D depression score was set as the dependent variable for the regression model, whereas the IAT overall score and six IAT subscale scores were set as the independent variables of the regression model.

## Results

### Participant demographics

The final sample of this study comprised 503 female junior college students. Statistics related to the participants’ demographic characteristics, depression, health-related behaviors, and Internet depression are presented in Table 1. Most of the participants were aged between 15 and 22 years (mean age = 17.30 years, SD = 1.34). Regarding smoking and alcohol drinking status, the majority of the students did not engage in either smoking (95.8%) or alcohol drinking (96.4%). Almost half of the students in the study sample (48.7%) were religious. With regard to the CES-D depression score, 242 (48.1%) of the students scored more than 16 points and therefore exhibited depressive tendencies. For the HPLP score, the mean scores of each item on the HPLP subscale were as follows (arranged from highest to lowest): interpersonal support behavior = 2.15 (±0.70); self-actualization = 1.78 (±0.71); nutritional behavior = 1.67 (±0.64); stress management behavior = 1.63 (±0.57); exercise behavior = 0.93 (±0.70); and health responsibility behavior = 0.69 (±0.59). Finally, regarding the IAT score, the results revealed that 32.6% of the participants exhibited no signs of Internet addiction, 52.7% of the participants exhibited a mild level of Internet addiction, and 13.9% of the participants exhibited a moderate-to-severe level of Internet addiction.

**Table 1:** Demographic, anthropometric, and lifestyle characteristics as well as scores on depression, Health Promoting Lifestyle Profile (HPLP) and level of Internet addiction.

	Total
	N = 503
**Age (mean ± SD)**	17.30 ± 1.34
**BMI (mean ± SD)**	20.50 ± 3.62
**Smoking habit (n,%)**	
**No**	482 (95.8%)
**Yes**	21 (4.2%)
**Drinking habit (n,%)**	
**No**	485 (96.4%)
**Yes**	18 (3.6%)
**Religion (n,%)**	
**No**	258 (51.3%)
**Yes**	245 (48.7%)
**CES-D (mean ± SD)**	16.23 ± 8.73
**0–15 (n,%)**	261 (51.9%)
**16–20**	102 (20.3%)
**21–30**	105 (20.9%)
**31–60**	35 (7%)
**HPLP scores (mean ± SD)**	
**Nutrition**	8.34 ± 3.18
**Health responsibility**	5.49 ± 4.72
**Self-actualization**	14.27 ± 5.70
**Interpersonal support**	12.93 ± 4.22
**Exercise**	3.74 ± 2.80
**Stress management**	14.64 ± 5.16
**Total scores**	59.41 ± 19.29
**IAT level (n,%)**	
**Normal**	164 (32.6%)
**Mild**	265 (52.7%)
**Moderate**	70 (13.9%)
**Severe**	4 (0.8%)
**IAT symptoms (mean ± SD)**	
**Total scores**	37.34 ± 12.00
**Salience**	7.58 ± 3.10
**Excessive Use**	10.22 ± 3.37
**Neglect of Work**	4.78 ± 1.86
**Anticipation**	3.64 ± 1.51
**Lack of Control**	5.66 ± 2.18
**Neglect of Social Life**	4.20 ± 1.71

BMI: Body mass index; CES-D: Center for Epidemiologic Studies-Depression; IAT: Internet Addiction Test; HPLP: Health Promoting Lifestyle Profile.

### Relationship between depression and health-related behaviors

Pearson's correlation coefficient (r) between the CES-D depression scores and the HPLP overall and subscale scores were -0.11 (*p* = 0.01), -0.20 (*p* < 0.01), -0.02 (*p* = 0.71), -0.16 (*p* < 0.01), -0.06 (*p* = 0.19), -0.01 (*p* = 0.92), -0.06 (*p* = 0.15) for the overall scores, nutrition subscale score, health responsibility subscale score, self-actualization subscale score, interpersonal support subscale score, exercise subscale score, and stress management subscale score, respectively. The regression analysis results for the CES-D and HPLP are presented in Table 2. The regression results indicate that only the HPLP overall score, nutrition subscale score, and self-actualization subscale score were significantly negatively associated to the CES-D depression score (*p* < 0.05–0.01). In other words, depression was lower in the students who exhibited more healthy behaviors, placed more emphasis on dietary health, and had higher levels of self-admiration and confidence toward life.

**Table 2:** Multiple regression analysis for identifying depression significantly related to health-related behaviors^†^.

	B	SE	OR (95% CI)	*p*
**HPLP scores**				
**Nutrition**	-0.55	0.12	-0.79, -0.31	<0.01
**Health responsibility**	-0.04	0.08	-0.21, 0.12	0.61
**Self-actualization**	-0.23	0.07	-0.37, 0.10	<0.01
**Interpersonal support**	-0.12	0.09	-0.30, 0.07	0.22
**Exercise**	-0.01	0.14	-0.28, 0.26	0.94
**Stress management**	-0.11	0.08	-0.26, 0.04	0.16
**Total scores**	-0.05	0.02	-0.09, 0.01	0.01*

^†^Controlled for age, body mass index (BMI), smoking and drinking habits, religion

*p < 0.05; B: regression coefficient; S.E.: standard error; OR: odds ratio; CI: confidence interval.

### Relationship between depression and Internet addiction

The data related to the demographic characteristics and CES-D depression scores of the three Internet addiction severity groups are presented in Table 3. The ANOVA results revealed that the three Internet addiction severity groups only differed significantly in terms of their CES-D depression scores (*p* < 0.01). According to the results of the Tukey post hoc tests, the moderate-to-severe Internet addiction group scored significantly higher on the CES-D depression score compared with the mild addiction group, and both the moderate-to-severe group and the mild group scored significantly higher on the CES-D depression score compared with the normal group.

**Table 3:** The demographic, anthropometric, and lifestyle characteristics as well as scores on depression in students with different degrees of Internet addiction.

	IAT level			*p*	Post Hoc
	Normal (a)	Mild (b)	Moderate to severe (c)		Tukey
**Age (mean ± SD)**	17.40 ± 1.35	17.28 ± 1.32	17.15 ± 1.38	0.39 ^b^^,^	
**BMI (mean ± SD)**	20.40 ± 3.96	20.57 ± 3.34	20.44 ± 3.86	0.67 ^b^^,^	
**Smoking habit**				0.31^a^^,^	
**No (n,%)**	156 (95.1%)	257 (97.0%)	69 (93.2%)		
**Yes (n,%)**	8 (4.9%)	8 (3.0%)	5 (6.8%)		
**Drinking habit**				0.96 ^a^	
**No (n,%)**	158 (96.3%)	256 (96.6%)	71 (95.9%)		
**Yes (n,%)**	6 (3.7%)	9 (3.4%)	3 (4.1%)		
**Religion**				0.85 ^a^	
**No (n,%)**	87(53.0%)	133(50.2%)	38(51.4%)		
**Yes (n,%)**	77 (47.0%)	132 (49.8%)	36 (48.6%)		
**CES-D (mean ± SD)**	12.88 ± 6.94	16.60 ± 8.44	22.32 ± 9.77	<0.01 ^b^^,^**	(c)>(b)>(a)

**p < 0.01

^a^ Significance of difference determined using Chi Square test

^b^ Significance of difference determined using ANOVA.

Pearson's correlation coefficient (r) between the CES-D depression scores and the IAT overall and six domain scores were 0.41 (*p* < 0.01), 0.52 (*p* < 0.01), 0.37 (*p* < 0.01), 0.30 (*p* < 0.01), 0.27 (*p* < 0.01), 0.35 (*p* < 0.01), 0.31 (*p* < 0.01) for the overall scores, salience, excessive use, neglect of work, anticipation, lack of control, and neglect of social life, respectively. The full results of the CES-D and IAT regression analysis are presented in Table 4. The regression analysis results revealed that the IAT overall score and six domain scores were all positively associated (*p* < 0.01) to the CES-D depression score, with or without controlling for the HPLP overall score. In other words, the higher an individual’s Internet addiction score was, the higher her depression level was.

**Table 4:** Multiple regression analysis for identifying depression significantly related to Internet addiction symptoms.

	B	SE	OR (95% CI)	*p*
**Model 1: IAT symptoms**				
**Total scores**	0.30	0.03	0.24, 0.36	<0.01
**Salience**	1.47	0.11	1.26, 1.69	<0.01
**Excessive Use**	0.96	0.11	0.75, 1.17	<0.01
**Neglect of Work**	1.47	0.20	1.08, 1.87	<0.01
**Anticipation**	1.59	0.25	1.10, 2.09	<0.01
**Lack of Control**	1.45	0.17	1.12, 1.78	<0.01
**Neglect of Social Life**	1.60	0.22	1.18, 2.03	<0.01
**Model 2: IAT symptoms**				
**Total scores**	0.29	0.03	0.23, 0.35	<0.01
**Salience**	1.46	0.11	1.24, 1.67	<0.01
**Excessive Use**	0.93	0.11	0.72, 1.14	<0.01
**Neglect of Work**	1.44	0.20	1.05, 1.84	<0.01
**Anticipation**	1.55	0.25	1.06, 2.04	<0.01
**Lack of Control**	1.41	0.17	1.07, 1.74	<0.01
**Neglect of Social Life**	1.58	0.22	1.17, 2.01	<0.01

Model 1: Controlled for age, body mass index (BMI), smoking and drinking habits, and religion.

Model 2: Controlled for age, body mass index (BMI), smoking and drinking habits, religion and health-related behaviors.

B: regression coefficient; S.E.: standard error; OR: odds ratio; CI: confidence interval.

## Discussion

After controlling for possible confounding factors, the results of the present study revealed that (1) depression is significantly associated to two health-related behaviors and (2) depression is significantly related to Internet addiction (and the six patterns of Internet addiction symptoms). These results indicate the possibility of alleviating depression in female adolescents through the promotion of health-related behaviors. Additionally, they indicate that depression could possibly worsen Internet addiction behavior. The analysis results presented in Table 1 show that almost half of the Taiwanese female students in this study exhibited depressive tendencies (48.1% ≥ 16), which is higher than among Australian female adolescents (31.8% ≥ 16; [45] but lower than among female adolescents in Hong Kong (62.05% ≥ 16 [24]. This is possibly due to cultural differences between East Asian and Western countries. Compared with parents and teachers from Western countries, parents and teachers (especially Chinese parents and teachers) from East Asia (e.g., Hong Kong and Taiwan) generally emphasize learning and academic achievement. They are willing to invest large amounts of resources (be they financial or temporal resources), and they often emphasize the role of recitation in the learning process and believe it helps to develop higher order thinking skills [46]. However, such emphasis can heighten the emotional stress (i.e., the fear of performing poorly or making mistakes) experienced by East Asian students. According to a study [1], 64.8% of Taiwanese adolescents cited academic stress as their top source of stress. By contrast, parents from Western countries generally provide a relatively free learning environment for their adolescents, and teachers from Western countries encourage their students to cultivate their creativity and imagination [46]. Studies have reported that depressive emotions can affect the development of female adolescents negatively; in the short term, depressive emotions can lead to suicidal ideation or behavior [47], and in the long run, depressive emotions can lead to early marriage or marital problems [48]. Overall, the results of this study highlight the need to seriously consider the emotional problems of female teenagers, and more research should be conducted to examine related issues in depth.

Regarding health behavior performance, the results presented in Table 1 show that participants performed the best in terms of interpersonal support behaviors and performed the worst in terms of health responsibility behaviors; these results highlight the importance of interpersonal relationships for female adolescents. Compared with male adolescents, female adolescents may value interpersonal relationships (i.e. friends) more, and the quality of interpersonal support they receive can affect their psychological [49] and physiological health [50]. A longitudinal study [51] revealed that receiving high-quality social support can help to lower the risk of future suicidal ideation in female adolescents. By contrast, the female adolescent participants in this study performed relatively poorly in terms of health responsibility behaviors, which is consistent with the results of previous studies [52, 53]. A possible explanation for this is that the adolescent participants were still too young to worry about or pay attention to their health. Therefore, they were less likely to regularly perform health responsibility behaviors such as checking their blood pressure and cholesterol levels regularly, participating in health education courses, and avoiding eating foods laden with preservatives [54]. With regard to Internet addiction, the results presented in Table 1 show that more than half of the participants in this study exhibited at least a mild level of Internet addiction, indicating that the Internet has become an indispensable necessity for female adolescents in modern society. The Internet facilitates the transfer of large amounts of information in a short time and enables interaction between people around the world. For teenagers, it serves as a platform for expressing their feelings and stress relief; when they face difficulties or negative emotions in real life, the Internet can provide a space for them to engage in self-regulation [55, 56]. However, over indulgence in the Internet can easily lead to the development of negative emotions such as depression and anxiety [26] and negatively affect academic performance and family relationships [57].

The results of the multiple regression analysis presented in Table 2 revealed significant correlations between depression, nutritional behaviors, and self-actualization. Regular healthy dietary behaviors (e.g., eating breakfast regularly and consuming fruits and vegetables) are beneficial for promoting emotion regulation [58] and reducing negative emotions in adolescents [59, 60]. According to a systematic review [61], adolescents who exhibit healthy dietary behaviors or consume high-quality diet have lower levels of depression and better mental health. Emotional regulation is influenced by different neurochemical pathways (e.g., the dopamine pathway) in the human body, and each pathway requires various types of nutrients to produce the metabolites needed by the neurotransmitters involved in the emotional regulation process [62]. Several studies [63, 64] have suggested that ingesting enough nutrients (e.g., eicosapentaenoic acid, docosahexaenoic acid) from the daily diet is beneficial for emotional improvement. Nevertheless, negative emotions (e.g., depression) can affect adolescents’ emotional regulation ability and heighten their risk of becoming obese due to junk food consumption [58]. Because the present work was a cross-sectional study, the results cannot be used to explain causal relationships between variables. Further studies are therefore necessary to provide supporting evidence of the relationship between dietary behaviors and depression.

The results of the present study also revealed that depression levels were lower in adolescents with higher self-actualization scores, which is consistent with the results of previous studies [65]. Adolescents with high self-actualization are likely to possess higher levels of self-admiration, possess higher levels of self-confidence, be more optimistic, and feel that life is meaningful and full of fun and challenges [66]. These adolescents are also likely to achieve Maslow's hierarchy of needs [67]. Compared with their peers, such teenagers are likely to exhibit better functional performance in life tasks such as gaining higher social status, making friends, and looking for spouses [68]. One study [69] found that the self-actualization status of college students was related to their academic orientation. In other words, university students who exhibited higher levels of self-actualization also exhibited better performance in terms of creative expression, reading for pleasure, and academic efficacy. Additionally, studies have reported that students who exhibit higher levels of self-actualization have higher sleep quality [70–72]. In short, a high level of self-actualization can possibly lower the occurrence of negative emotions. Adolescence is the peak onset period for clinical depression. Thus, reducing depression risk through health-related behaviors is a research direction worthy of further exploration.

The analysis results presented in Tables 3 and 4 confirmed the relationship between Internet addiction and depression. These results are consistent with the findings of past studies [16, 23–25] that higher levels of Internet addiction are associated with higher levels of depression in adolescents. The results of the present study further revealed that all six patterns of symptom complaint in the IAT are significantly related to depression level. In other words, female adolescents with depression may also face social and life problems caused by their Internet usage. Adolescents with depressive moods typically have poorer social skills than those without such moods; additionally, they also typically harbor negative expectations while interacting with others, and they may interpret certain ‘micromessages’ as rejections [73]. These interaction tendencies may cause such adolescents to have difficulties in forming real-world relationships, and they subsequently may have less chances than others to learn social skills, forming a vicious cycle of poor social functioning and depressive mood [73]. Compared with face-to-face communication, online communication holds many advantages for adolescents, because it is generally anonymous, unrestricted by time and place, less focused on one’s outer appearance, and provides individuals with an area where they can lead social interactions [74, 75]. Additionally, adolescents believe that online communication protects them from “rejections” by others. The aforementioned reasons perhaps explain why adolescents can become addicted to the online world [55]. Depression has been reported to be related to unhealthy addictive behaviors; individuals may try to lower their anxiety and reduce their negative emotions though addictive behaviors [76]. For teenagers with depression, their attempts to regulate their emotions through Internet usage may increase the risks of developing Internet addiction [77]. However, excessive Internet use may disrupt daily functioning and produce various negative consequences, such as a lack of time management, difficulty in concentrating on academic tasks, poor emotional regulation, and a refusal to interact with others (Table 4). Because the Internet provides many stimulating activities, including online games, online shopping, and online communication; individuals with depression may fully engage themselves in the online to try and avoid negative emotions [55].

Although the influence of confounding factors was controlled for in this study, the study still had several limitations. First, measurement of depressive emotions was conducted using a self-report scale, namely the CES-D. Although the CES-D has been extensively used as a tool for measuring depressive emotions in adolescents and has been found to possess good reliability and validity [38], the results of the CES-D cannot be considered as a diagnosis of clinical depression. Second, this study was a cross-sectional study, and therefore the results cannot be taken as evidence of causal relationships between the variables. Further studies are required to examine the causal relationships between depression and health-related behaviors. Third, the possibility that the participants were facing academic-related stress during the data collection period cannot be discounted, and this could have affected the validity of the results. Lastly, all participants in this study were recruited from a single junior college in southern Taiwan, and this limits the generalizability of the findings. Nevertheless, even with these limitations, the results can still serve as a useful reference for parents and educators caring for female adolescents. Future studies recruiting both genders are warranted to explore the gender differences.

## Conclusion

In conclusion, the results of the current study confirmed the relationships between depression, health-related behaviors, and Internet addiction, indicating that cultivation of health-related behaviors may help in lowering depressive symptoms. Teenagers with depression are at higher risk of developing Internet addiction, which can affect daily functioning. Therefore, developing effective strategies to alleviate depressive emotions in female adolescents is crucial.

## Supporting information

S1 Data (SAV)
